# Low-level laser therapy in the management of muscle fatigue caused after long Endodontic procedure

**DOI:** 10.4317/jced.60369

**Published:** 2023-05-01

**Authors:** Trupti-Vijay Gaikwad, Anuj-Paul Maini, Sukanya Das, Subhadeep Gupta, Arunima Sarma, Ashutosh Dighe

**Affiliations:** 1 Post Graduate Student, Dept. of OMR, Dr. D Y Patil Dental College, Dr. D Y Patil Vidyapeeth, Pune, India; 2 Professor, Dept. of OMR, Dr. D Y Patil Dental College, Dr. D Y Patil Vidyapeeth, Pune, India; 3 Assistant Professor, Dept. of OMR, Dr. D Y Patil Dental College, Dr. D Y Patil Vidyapeeth Pune, India; 4 Assistant Professor, Dept. of OMR, Vananchal Dental College and Hospital, Garhwa, India

## Abstract

**Background:**

The masticatory muscles may undergo fatigue due to prolonged mouth opening during the endodontic procedures. Low-level laser can be used to treat muscle fatigue due its capacity to produce reactive oxygen species and improve function of mitochondria. Aim: To determine the effectiveness of low-level laser therapy in the management of masticatory muscle fatigue caused after long endodontic procedure under Local anesthesia.

**Material and Methods:**

44 patients complaining of reduced mouth opening and pain while mouth opening, after long endodontic therapy were considered for the study and were randomly allocated into study and control group. In the study group, low-level laser was applied while patients of control group didn’t receive any therapy. In the study group, Visual analogue scale (VAS) score of pain was taken after endodontic therapy, immediately after laser therapy and 4 hours after endodontic therapy. Mouth opening of the patients was measured, before and after endodontic procedure and immediately after laser therapy. In the control group, VAS score of pain was recorded immediately after endodontic therapy and 4 hours after endodontic therapy. Statistical analysis used: ANOVA test and un-paired t-test was used for the data analysis.

**Results:**

When both groups were compared, a statistically significant (*P*=0.0000) reduction with fatigue was found.

**Conclusions:**

The low-level laser can be a useful procedure immediately post long endodontic procedure causing masticatory muscle fatigue. Hence, this therapy can be considered as an add-on therapeutic procedure along with prolonged endodontic appointments to relieve the patient from the discomfort.

** Key words:**Muscle fatigue, Masticatory muscles, Low-level laser therapy.

## Introduction

The mandibular condyle and the fossa of the temporal bone form the gliding joint called the Temporo-mandibular Joint (TMJ). Masticatory muscles cause movement of this joint which include the masseter, medial pterygoid, lateral pterygoid and temporal muscles (1).

TMD represents a wide range of jaw conditions including disorders of the temporo-mandibular joint, masticatory muscles, nervous system and behavior (2). It is believed that the inflamed lateral pterygoid muscle is very painful and shows abnormal patterns of muscle activity in TMD patients. It plays a significant role in mouth opening (3).

Root Canal Treatment (RCT), a routine procedure has been performed since many years following traditional protocols (4). Persistent pain following RCT is a very common event. The most common non-odontogenic reason for pain is TMD with involvement of muscles of mastication (5). Root canal therapy, by an inexperienced student is usually a lengthy treatment, especially for multi-rooted teeth. While performing such procedures, the masticatory muscles and articular ligaments are stretched for a longer time period, which may cause spasm in muscles, pain and discomfort while opening mouth and chewing, limited opening of mouth (6). Performing a motor task for prolonged period of time can cause motor fatigue in the masticatory muscles. There is decline in ability of a person to exert force. It is developed after the onset of the sustained physical activity gradually, like wide opening of mouth (7). Prolonged mouth opening due to the long Root canal Treatment causes fatigue of the lateral pterygoid muscles causing pain in the TMJ region especially among individuals aged above 50 years (8,9).

At present, there are no recommended options of treatment of muscle fatigue. However, some synthetic products like caffeine and amphetamine, natural products like rhodiola rosea and American ginseng and few nutritional supplements like vitamins, minerals and creatine, have shown positive effects in various studies (10).

In order to improve muscle performance and delay the process of fatigue in the muscle, therapy of Low-level laser has been widely administered. There have been reports of improvements in training of strength, reduction of inflammation and exhaustion recovery in the studies involving human subjects (11). Studies of the mechanisms involved show that low-level laser therapy (LLLT) decreases oxidative stress and production of reactive oxygen species. It also improves function of mitochondria, and causes stimulation of respiratory chain of mitochondria, synthesis of Adenosine Triphosphate (ATP), and microcirculation (12). And Blood flow also plays an important role in recovery from fatigue of muscle fiber (9).

All these effects give the rationale for testing LLLT, whether it can be helpful in reducing muscle fatigue caused after long endodontic procedure.

## Material and Methods

This study involving human participants was reviewed and approved by the Institutional Ethics Committee (Ref No. DYPDCH/IEC/123/121/19 dated 25/09/2019). Written informed consent for participation was taken. The study was conducted within the framework of declaration of Helsinki in Department of Endodontics including 44 patients of varying age groups who were having muscle fatigue after one hour long Endodontic procedure under local anesthesia. Male-to-Female ratio was 13:7. There were no drop outs from the study.

Inclusion criteria.

1. Patients aged between 18-40 years.

2. Patients who were ready to give informed consent.

3. Patients undergoing Root canal treatment procedure >1 hour with respect to maxillary molars 16 and 26 under local anesthesia by the same post-graduate student.

Exclusion Criteria.

1. Patients with symptomatic deep carious lesions, periapical pathologies and odontogenic infections among teeth other than the tooth undergoing RCT.

2. Cases with oral/dental congenital abnormalities (Specific), neoplastic conditions and those with a recent history of acute facial trauma.

3. Patient with underlying systemic illness (Rheumatoid Arthritis or joint disorders)

4. Patients with history of TMDs or history of treatment for TMDs.

5. Patients with parafunctional habits causing pain.

6. Partially edentulous patients with pain due to imbalance.

Sample size calculation.

Sampling technique: Convenience Sampling

With effect size= 0.40

Alpha(α) = 0.05

Power= 0.80

Total Sample size- n = 22

Using G power 3.1

According to the sample size calculation, the control will also be taken as 22

Therefore, total sample size= 44

Case selection.

A detailed Case history of 44 patients, who were scheduled for more than 1 hour long endodontic procedure with respect to 16 and 26 and who satisfied the above-mentioned inclusion and exclusion criteria was recorded. The patients considered for the study were distributed randomly by lottery method into two groups, study and control group with 22 patients in each group. All the patients under-went the procedure of Root canal therapy under local anesthesia.

Treatment Protocol.

According to Roger *et al*. (13), muscle fatigue is a symptom that causes reduction in the ability of muscles to perform over time. Considering which, we have stated the discomfort in opening mouth after prolonged mouth opening as muscle fatigue.

**Figure 1:**
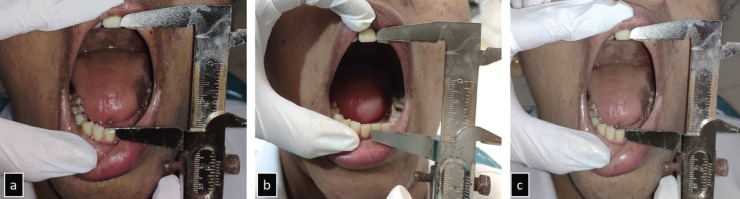
a) Mouth opening before endodontic procedure. b): Mouth opening after endodontic procedure. c): Mouth opening after low-level laser therapy.

In a study by Mehmet *et al*. (14), the fatigue severity in the knee muscles was assessed using visual analogue scale (VAS), which ranged from 0 mm to 100 mm. We have also used a similar 10-mm scale (VAS), which ranges from 0 mm (no fatigue) to 10 mm (extreme fatigue) for the assessment of masticatory muscle fatigue. Immediately after the completion of the endodontic procedure, all the 40 patients were asked to fill a VAS proforma regarding the discomfort in mouth opening. In addition, mouth opening before and after the endodontic therapy and after laser therapy was also measured in study group using Vernier caliper to determine the disability caused due to fatigue (Fig. 1a-c).

**Figure 2:**
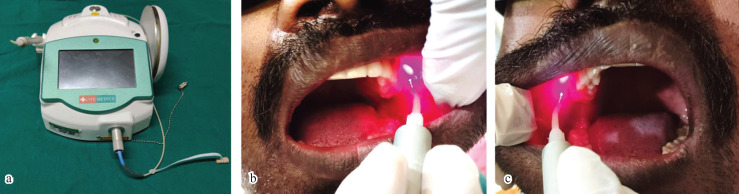
a) Diode laser Unit. b,c): Low-level laser therapy on left and right side intraorally.

Huge number of studies have been performed to treat myalgia and pain associated with TMDs using low level laser therapy (15-17). However, no study has ever been performed to treat the masticatory muscle fatigue caused due to endodontic procedure. 22 patients in the study group underwent the Low-level laser therapy with diode laser (Litemedics, 940nm wavelength, 0.6W intensity, non-contact, non-focused mode) (Fig. 2a). For palpation of lateral pterygoid muscle, we can place our index finger or tip of our little finger in the vestibular area adjacent to the maxillary third molar (18). Therefore, the laser was applied to the lateral pterygoid muscle near the point of its palpation for 1 minute on each side (Fig. 2b,c). Other 22 patients in the control group did not undergo any therapy.

The patients in the study group were asked to fill the VAS proforma again immediately after the low-level laser therapy. Mouth opening was also measured immediately after the laser therapy in study group (Fig. 1c). 4 hours after the endodontic therapy/laser therapy, patients were again asked to fill the VAS proforma in study and control groups.

There were potential sources of bias like the pain of odontogenic origin which was eliminated due to the administration of local anesthesia. Muscle fatigue is more common in individuals with old age (8) therefore, patients above age of 40 years were not included in the study. Tooth no. 16 and 26 were only considered, to eliminate the bias due to the variability in the location and morphology of the teeth. Patients with a history of Parafunctional habits, TMDs and underlying systemic illness related to the joints and muscles were also excluded from the study.

Statistics:

Data obtained was tabulated and statistical analysis was performed. Microsoft Excel data analysis was used for statistical analysis. Descriptive statistics, ANOVA test and un-paired t-test was applied to analyze the data obtained from the study.

## Results

Table 1 represents the descriptive data regarding the VAS score at different time intervals in study group and control group respectively. The descriptive data regarding the mouth opening is shown in Table 2, Table 3 represents the comparison of VAS score and mouth opening at different intervals between the two groups.

**Table 1:**
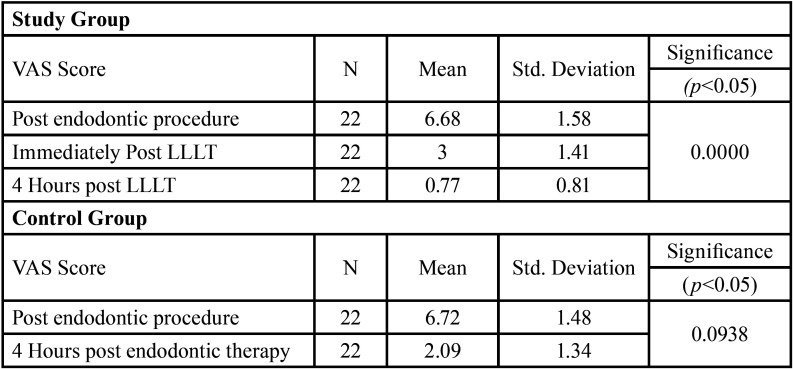
Descriptive data regarding VAS score at different time intervals in study group and control group.

**Table 2:**
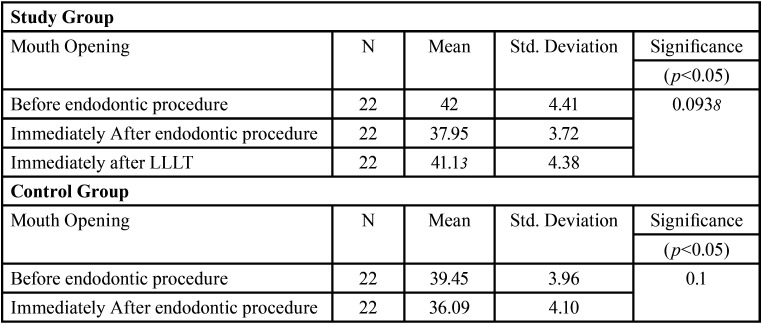
Descriptive data regarding Mouth opening at different time intervals in study group and Control group.

**Table 3:**
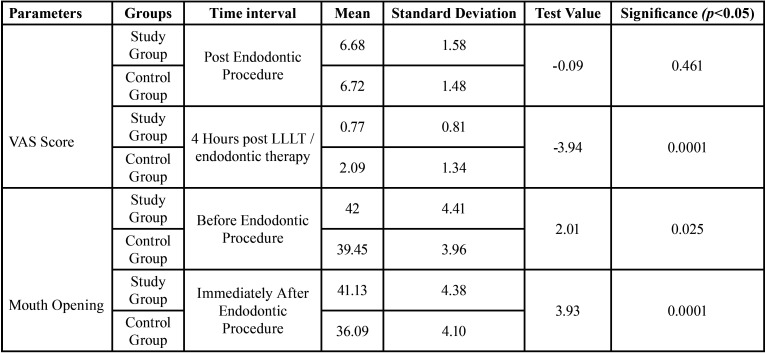
Difference between VAS score and mouth opening at different intervals between the two groups.

## Discussion

Muscles used extensively show a decline of performance progressively which usually recovers after a period of prolonged rest. This reversible phenomenon is denoted as muscle fatigue (19). There is a significant literature describing the mechanism involved in muscle fatigue and other processes associated with it (20). Mitochondria are recruited to supply the demand of Oxygen. The process of oxidative phosphorylation occurs in the mitochondria which results in oxygen consumption and Adenosine Triphosphate (ATP) production. During this process, reactive oxygen species (ROS) are generated. The rate of production of ROS depends upon the intensity of work. The oxidation of critical cellular proteins, like the Na+-K+ pump, Dihydropyridine receptor (DHPR), myofilaments, and Ryanodine receptor 1 (RYR-1) is caused by the ROS which plays a vital role in muscle fatigue. The process of oxidation of ROS can assist in the development of low-frequency fatigue and inhibit the release of Ca2+ from Sarcoplasmic reticulum (21).

Muscle fatigue can cause swelling, pain, loss of muscle strength and power loss, reduction in range of motion, muscle soreness which is delayed in onset and impaired recovery which may result in impairment of performance. The muscle strength is reduced by 20-50% immediately after any task which may take 2 to 7 days to recover fully (22). In the present study, the cause of masticatory muscle fatigue is the prolonged mouth opening task that is being performed due to undergoing prolonged endodontic procedure.

Studies have shown that there are various treatment options, like medication including non-steroidal anti-inflammatory drugs (NSAIDs), physical therapies such as massage and stretching exercises and other modalities like ultrasound and antioxidants have minimal effects in terms of reduction of muscle fatigue (23). Currently, there are no official recommendations for the treatment of fatigue of the muscles (10). Low-level laser therapy treats pain, promotes healing and also shows interaction with the biological tissues. Moreover, LLLT has also been used to reduce muscle fatigue in various clinical trials and experimental models. Muscle fatigue can be reduced if LLLT is applied before or after intense exercise (24). LLLT application is related to mitochondrial function. The light interacts with the mitochondria promoting cellular changes that may contribute to delay muscle fatigue. LLLT prevents cellular apoptosis; stimulates mitochondrial activity; increases cell recruitment, proliferation, and renewal; and modulates cell metabolites providing relief from muscle fatigue (21).

Shobha *et al*. (25), evaluated the efficacy of therapy with low-level laser in reducing the TMJ pain. Significant reduction in pain and improvement in mouth opening was observed. A clinical trial had also been registered by Greice *et al*. (11), for the evaluation of the effectiveness of therapy with Low-level laser on the masticatory muscles. However, our study evaluated the effectiveness of therapy with low-level laser after long endodontic procedure.

44 patients had been randoml.y divided into study group and control group with 22 patients in each group. Out of the 22 patients in study group, 9 were females and 13 were males. The control group consisted 12 males and 10 females. The results show that the mean value of VAS score has significantly decreased in both study and control group but the decline is more in the study group after 4 hours with the mean of 0.77±0.81 while the mean of control group was 2.09±1.34. The mouth opening has also reduced in both study and control groups, with mean score being 37.95±3.72 and 36.09±4.10 respectively, immediately after endodontic therapy but the mouth opening has shown an increase in study group immediately after laser therapy with a mean score of 41.13±4.38. ANOVA test was used to assess statistical difference within the group at different time intervals (immediately after endodontic therapy, immediately after low level laser therapy and 4 hours after therapy with low-level laser / Endodontic therapy). A statistically significant difference was seen within the study group (*P* = 0.00) and control group (*P* = 0.00) when the VAS score was compared. The difference was found to be non-significant statistically, in both study group (*P* = 0.0938) and control group (*P* = 0.1), when the mouth opening was compared within the group. To compare the difference between the two groups (Study and Control), an unpaired t-test was used for two independent samples of 4 hours post LLLT and 4 hours post endodontic therapy. It was found that the difference is significant when both the groups are compared (*P* = 0.0001). Hence, the therapy with low-level laser can be considered effective in reducing the masticatory muscle fatigue after long/prolonged endodontic procedure.

Many studies are being performed on low-level laser to evaluate its effect on muscle fatigue. In a study (26), therapy with low-level laser was used to delay the process of skeletal muscle fatigue. The effects of red and infrared LLLT on skeletal muscle fatigue was compared. In another study (27), the effects of different LLLT doses on performance of cyclists in time-to-exhaustion tests was investigated. It was seen that, LLLT increased time to exhaustion in competitive cyclists. Our study is first of its kind. No study has been done on the evaluation and treatment of masticatory muscle fatigue. However, studies involving a larger sample size is necessary for more significant results. Studies involving muscles of mastication which may undergo fatigue during day to day activities is necessary. More trustful analysis of the biochemical markers which are increased due to the fatigue and electromyographic analysis of the masticatory muscles is necessary.

## Conclusions

This study with a small sample size was done to evaluate the effectiveness of therapy with low-level laser on masticatory muscle fatigue after long endodontic procedure. It was seen that the study group was more effective in reducing the masticatory muscle fatigue. The findings indicate that the masticatory muscles are undergoing fatigue due to the prolonged mouth opening task that is being performed and low-level laser therapy relieves the patients from the symptoms of fatigue. Therefore, instead of cutting down the appointments into short ones, long appointments can be scheduled if the muscle fatigue is being treated with low-level laser.
